# Association between weight-adjusted waist index and testosterone deficiency in adult American men: findings from the national health and nutrition examination survey 2013–2016

**DOI:** 10.1186/s12889-024-19202-5

**Published:** 2024-06-24

**Authors:** Dalu Liu, Yuanyuan Li, Nuo Ji, Wei Xia, Bo Zhang, Xingliang Feng

**Affiliations:** 1 Department of General Surgery, The Affiliated Chuzhou Hospital of Anhui Medical University (The First People’s Hospital of Chuzhou), Chuzhou, Anhui China; 2 Department of General Surgery, The Third Affiliated Hospital of Soochow University, Changzhou, Jiangsu China; 3 Department of Urology, The First People’s Hospital of Changzhou, Changzhou, Jiangsu China; 4 Department of Urology, The Third Affiliated Hospital of Soochow University, Changzhou, Jiangsu China

**Keywords:** Weight-adjusted Waist Index, Testosterone deficiency, Obesity, Testosterone level, NHANES, Cross-sectional study

## Abstract

**Background:**

Testosterone deficiency (TD) and obesity are globally recognized health concerns, with a bidirectional causal relationship between them. And a newly discovered obesity indicator, the Weight-Adjusted-Waist Index (WWI), has been proposed, demonstrating superior adiposity identification capability compared to traditional body mass index (BMI) and waist circumference (WC) indicators. Therefore, we present the inaugural investigation into the associations of WWI with total testosterone levels and the risk of TD.

**Methods:**

Data restricted to the National Health and Nutrition Examination Survey (NHANES) between 2013 and 2016 were analyzed. Only males aged > 20 years who completed body measures and underwent serum sex hormone testing were potentially eligible for analysis. Weighted multivariable linear regression and logistic regression analyses were employed to investigate the relationships between WWI and total testosterone levels, and the risk of TD, respectively. Smooth curve fittings and weighted generalized additive model (GAM) regression were conducted to examine the linear relationship among them. Additionally, subgroup analyses with interaction tests were performed to assess the stability of the results.

**Results:**

Finally, a total of 4099 participants with complete data on testosterone and WWI were included in the formal analysis. The mean age of study participants was 46.74 ± 0.35 years with a TD prevalence of 25.54%. After adjusting all potential confounders, the continuous WWI displayed a negative linear relationship with total testosterone levels (β=-61.41, 95%CI: -72.53, -50.29, *P* < 0.0001) and a positive linear relationship with risk of TD (OR = 1.88, 95%CI: 1.47, 2.39, *P* < 0.0001). When WWI was transformed into quartiles as a categorical variable, participants in Q4 exhibited lower total testosterone levels (β=-115.4, 95%CI: -142.34, -88.45, *P* < 0.0001) and a higher risk of TD (OR = 3.38, 95% CI: 2.10, 5.44, *P* < 0.001). These associations remained stable in subgroup analyses without significant interaction (all P for interaction > 0.05).

**Conclusions:**

This investigation firstly unveiled a negative linear association between WWI and total testosterone levels, coupled with a positive linear relationship with the prevalence of TD in U.S. male adults aged 20 years and older. Further studies are needed to validate the potential utility of WWI for the early identification and timely intervention of TD.

**Supplementary Information:**

The online version contains supplementary material available at 10.1186/s12889-024-19202-5.

## Background

Testosterone is an indispensable male sexual hormone principally produced by the testicular Leydig cells and is primarily regulated by negative feedback of the hypothalamic-pituitary-gonadal axis (HPGA) [1]. Maintaining normal testosterone levels is crucial for regulating a range of males’ physiological functions, including male reproduction and sexual functions, cardiovascular health, metabolic processes, and psychological cognitive functions [2–6]. However, testosterone deficiency (TD) is an exceedingly common condition in males afflicting approximately 30% of men aged 40 to 79 years, whose incidence rose with aging and some prevalent medical conditions, such as obesity, diabetes, and hypertension [7]. It’s worth noting that besides sexual symptoms like decreased libido, erectile dysfunction, and difficulty in achieving orgasm, TD can also lead to a range of serious nonsexual symptoms including cardiovascular diseases, decreased bone mineral destiny, depression, and obesity, all of which could adversely lower male’s quality of life [1, 8]. Therefore, TD has emerged as an increasingly concerning global health issue [9].

Obesity is another globally recognized health issue that is increasingly drawing attention, with the projected global prevalence expected to reach 18% in men and surpass 21% in women by 2025 [10]. More critically, the latest forecasts indicated that by 2023, half of the adult population will be categorized as obese [11]. Obesity has consistently been identified as a modifiable risk factor for the onset of TD [12, 13]. Furthermore, the relationship between obesity and TD has been demonstrated to be bidirectional, creating a detrimental cycle [14, 15]. Previous studies have explored the relationship between body mass index (BMI) and testosterone levels, indicating that obese individuals tend to have lower testosterone levels [16]. Notably, compared to individuals with a BMI < 18.5 kg/m^2^, those with a BMI > 35 kg/m^2^ have a significantly increased risk of testosterone deficiency, with an odds ratio (OR) of 2.51 and a 95% confidence interval (CI) of 1.19 to 5.32 [17]. However, as research has progressed, BMI as an indicator of obesity has been increasingly questioned due to its inability to differentiate between lean body mass and fat body mass [18]. With the evolution of understanding obesity, visceral adiposity combined with central obesity are gaining increased attention from researchers due to its heightened relevance to poor metabolic characteristics [19]. Consequently, researchers have attempted to use waist circumference (WC) to assess visceral fat and abdominal obesity in order to explore their relationship with testosterone levels [20]. Despite the proven associations of WC with testosterone levels, the strong association between WC and BMI makes researchers skeptical of WC’s effectiveness as a reliable indicator of visceral obesity [21]. As a result, researchers continue to seek novel indicators capable of accurately assessing visceral adiposity, which are imperative to thoroughly elucidate the relationship between visceral obesity and TD. Furthermore, these indicators should be straightforward, time-efficient, cost-effective and applicable across various clinical settings, in stark contrast to technologies such as densitometry (dual-energy X-ray absorptiometry, DXA), magnetic resonance imaging (MRI), computed tomography (CT), and mechanical methods, which are characterized by technical complexity, time-consuming, and high-cost [22].

A study by Park et al. [23]. was the first to propose a novel adiposity index called weight-adjusted-waist index (WWI), designed for assessing central obesity, with the formula WC/weight^1/2^. From the calculation formula of WWI, it could be concluded that WWI standardizes waist circumference (WC) for body weight, which allows extraction the benefits of WC while attenuating the correlation with BMI, thus primarily reflecting weight-independent centripetal obesity [24]. Subsequent studies have further confirmed the superiority of the Weight-to-Height Index (WWI) in accurately assessing visceral obesity compared to BMI and WC across a series of metabolic diseases [25–27]. Nevertheless, to date, the correlation between TD and WWI remains unclear. Additionally, whether the strength of the association between WWI and TD is greater than that between BMI, WC, and TD is also unknown. To address this knowledge gap and provide a more profound understanding of the relationship between visceral obesity and TD, we conducted this population-based study to investigate the correlation between WWI and TD, utilizing data from the National Health and Nutrition Examination Survey (NHANES).

## Materials and methods

### Survey description and study population

The NHANES is a continuous research program managed by the National Centre for Health Statistics (NCHS). Conducted in a two-year cycle, the NHANES aims to evaluate the nutritional and overall health of individuals of all ages in the U.S. population through interviews, physical examinations, and laboratory examinations. A sophisticated multistage probability design was employed to obtain a nationally representative sample of non-institutionalized United States residents. The NHANES study protocols were reviewed and approved by the Research Ethics Review Board of the NCHS, and all survey participants provided the written informed consent.

**Fig. 1:**
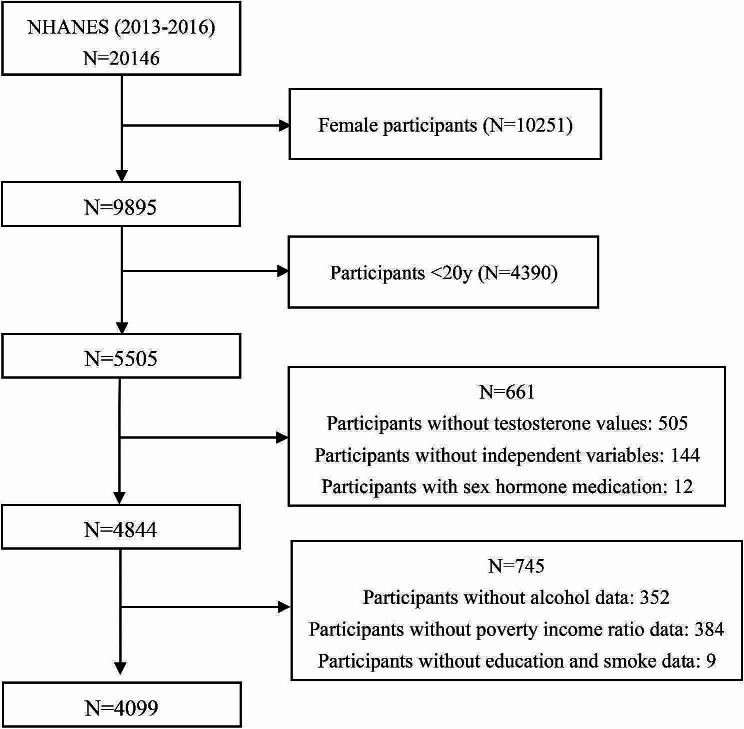
Flow chart of study population selection process. NHANES, National Health and Nutrition Examination Survey

For this research, the data were restricted to the continuous data cycles of 2013–2014 and 2015–2016. All men aged 20 years who completed body measures and underwent sex hormone testing were selected in the cohort. Participants without data on sex hormones and body measures were excluded. A total of 4099 participants were included after further exclusion of men who were taking sex hormone medication or had incomplete data on potential covariates. The detailed sample selection process was displayed in Fig. 1.

### Exposure variable and outcomes

The WWI (cm/√kg) was designed as the exposure variable, and the results for each participant were rounded to two decimal places. The WWI was calculated by the formula: WC in centimeters divided by the square root of weight (kg) [WWI (cm/√kg) = WC/√Weight)] [23]. WWI has shown a strong positive correlation with total body fat percentage, total abdominal fat area, and visceral fat area, while displaying a negative correlation with appendicular skeletal muscle mass and appendicular lean mass [28–30]. The anthropometry examinations of WC and weight were conducted in mobile examination center by trained health technicians under controlled conditions. Weight was measured using a digital scale accurate to the nearest 0.1 kg, and the WC was measured using a retractable steel measuring tape, also accurate to nearest 0.1 cm. During weighing, each subject wore a standard MEC examination gown, stood at the center of the digital scale, with hands at their sides and eyes looking straight forward. When measuring WC, the process began by locating the iliac crests through bilateral palpation. A horizontal line was then drawn just above the uppermost lateral border of the right ilium. Subsequently, the right midaxillary line was drawn. The measuring tape was positioned in the horizontal plane at the point where the two lines intersected. Finally, measurements were taken at the end of the individual’s normal expiration. The comprehensive procedure, covering protocols, equipment, and quality control, is available on the official NHANES website.

(https://www.cdc.gov/nchs/nhanes/index.htm). In our analysis, WWI was utilized both as a continuous variable and categorical variable based on quartiles.

The primary outcome of our analysis were the associations of WWI with total testosterone level and the occurrence of TD. Total testosterone levels were measured using isotope dilution liquid chromatography tandem mass spectrometry (ID-LC–MS/MS) at a single time point in the morning following an overnight fast, based on the National Institute for Standards and Technology’s (NIST) reference method. All sex steroid hormones were measured at Boston Children’s Hospital (Boston, MA, USA) by laboratory technicians blinded to participant characteristics. Similarly, details regarding the NHANES laboratory methodology for testosterone determination can be found on the official NHANES website. (https://www.cdc.gov/nchs/nhanes/index.htm). According to American Urological Association guidelines on TD, the TD was defined as a total testosterone level below 300ng/dL, measured on two separate occasions in the morning [31].

### Selection of covariates

The study identified and adjusted potential covariates based on published studies on WWI and factors pertaining to testosterone level and TD. These selected covariates encompassed demographic characteristics such as age (20-40y/40-60y/>60y), race/ethnicity (Mexican American/Non-Hispanic White/Non-Hispanic Black/Other Race), marital status (Solitude/ Cohabitation), educational level (Less than high school/High school/ More than high school), body mass index (BMI), and poverty income ratio (PIR) (< 1/≥1). These demographic characteristics were primarily obtained through interviews and physical examinations. Additionally, important health risk factors, including smoking status, alcohol consumption, hypertension and diabetes, were also considered. The smoking status were categorized into three groups as never smoker, former smoker, and current smoker, based on theirs answers to questions regarding whether smoked at least 100 cigarettes in their life time and if they were currently smoking cigarettes? Never smokers were defined as those who answered no to the both questions, while current smoker were identified as those who answered yes to the both questions. The remaining participants were categorized as former smokers. Participants who reported consuming at least 12 alcohol drinks per year were categorized as drinkers; otherwise, they were classified as non-drinkers. Participants with a diastolic blood pressure ≥ 90mmHg, a systolic blood pressure ≥ 140mmHg, a self-reported hypertension, the prescription of hypertension-lowering medication was considered hypertensive. Diabetes was defined as self-reported diagnosis, current use of insulin or diabetes pills, or meeting criteria such as a hemoglobin A1c level ≥ 6.5%, fasting plasma glucose level ≥ 126 mg/dl, or a plasma glucose level ≥ 200 mg/dl at 2 h after oral glucose tolerance test (OGTT). Prediabetes was determined by the absence of diabetes, with criteria including a hemoglobin A1c level between 5.7% and 6.4%, a fasting plasma glucose level ranging from 100 mg/dl to 125 mg/dl, or a 2-hour plasma glucose level after OGTT between 140 mg/dl and 199 mg/dl.

### Statistical analyses

Given the sample weighting utilized in the NHANES complicated multistage cluster survey design, all statistical analyses of the present study were conducted by incorporating appropriate sampling weights, strata, and primary sampling units. The continuous variables were expressed as weighted mean ± standard error (SE), and the categorical variables were presented as weighted percentage and SE. To compare baseline characteristic differences across different WWI quartiles, survey-weighted linear regression and survey-weighted Chi-square test were utilized for continuous and categorical variables, respectively. Two regression analyses were employed in our analyses: a multivariate linear regression analysis was utilized to assess the relationship between WWI and total testosterone levels, with results presented as β (95% CI); and a multivariate logistic regression analysis was conducted to evaluate the association between WWI and TD, with the results presented as the OR (95% CI). In both types of regression analyses, unadjusted and multivariable adjusted models were employed, with WWI treated as both a continuous variable and a categorical variable. In the multivariable regression models, adjustments were initially made in Model 1 for age, race, education, and PIR. Subsequently, in Model 2, additional adjustments were introduced for the remaining variables based on Model 1, including BMI, marital status, smoking, alcohol consumption, hypertension, and diabetes. Trend tests were performed using weighted linear regression when WWI was treated as categorical variables based on its quartiles in the models.

Smooth curve fitting and generalized additive models were employed to investigate whether there was a linear relationship between WWI and total testosterone levels, as well as the prevalence of TD. We also employed stratified multivariate regression analyses to conduct a subgroup analysis, aiming to explore the relationships of WWI with total testosterone level and TD in specific subgroups. Additionally, stratified analyses were further conducted when WWI was transformed into categorical variables (Q1-Q4). To assess heterogeneity among different subgroups, including age, BMI, diabetes, and hypertension, interaction terms were evaluated using the log-likelihood ratio test. Considering the multiple comparisons in our statistical analysis, we applied the Bonferroni correction to the p-values. The adjusted threshold for statistical significance is set at *P* < 0.005. All statistical analyses were performed using R software (Version 4.0.2) and the R package (http://www.R-project.org, The R Foundation) (23). Moreover, the EmpowerStats program (http://www.empowerstats.com, X&Y Solutions, Inc., Boston, MA, USA) significantly contributed to our study.

## Results

### Participants characteristics at baseline

Table 1 displays a detailed comparison of the baseline characteristics among the 4099 enrolled males in NHANES 2013–2014 and 2015–2016. The study population was categorized into quartiles of WWI, denoted as Q1 (8.54–10.34 cm/√kg), Q2 (10.34–10.90 cm/√kg), Q3 (10.90–11.44 cm/√kg), and Q4 (11.44–12.96 cm/√kg). The mean age of participants was 46.74 ± 0.35 years, demonstrating a statistical increase with higher WWI quartiles. The mean total testosterone level was 420.22 ± 4.29 ng/dL, with quartile 4 (340.68 ± 6.51 ng/dL) exhibiting lower testosterone levels than those in quartile 1 (508.01 ± 7.59 ng/dL). Likewise, the prevalence of TD also rose with a higher WWI (Q4: 43.31%, Q3: 28.80%, Q2: 21.78%, Q1: 11.67%), averaging at 25.54%. Participants in WWI quartile 4 were more likely to be obese but less likely to be Non-Hispanic Black or have an education level beyond high school. Furthermore, a higher prevalence rate of hypertension and diabetes was observed in WWI quartile 4 compared to quartile 1 (Hypertension: 60.27% vs. 18.33%; Diabetes: 33.66% vs. 2.91%). Intriguingly, no statistically significant difference in alcohol consumption was witnessed among the groups.

**Table 1:** Baseline characteristics of participants from NHANES 2013–2016 study by WWI quartiles, weighted

WWI	Total	Quartile 1	Quartile 2	Quartile 3	Quartile 4	*P* value
		8.54–10.34	10.34–10.90	10.90-11.44	11.44–12.96	
Participants number	4099	1025	1024	1027	1023	
Age, years	46.74 ± 0.35	34.89 ± 0.62	44.97 ± 0.55	51.41 ± 0.61	58.12 ± 0.65	< 0.0001
BMI, kg/m^2^	29.00 ± 0.15	24.63 ± 0.16	28.28 ± 0.19	30.23 ± 0.23	33.82 ± 0.25	< 0.0001
WC, cm	102.38 ± 0.42	87.20 ± 0.45	99.69 ± 0.42	107.22 ± 0.51	118.72 ± 0.57	< 0.0001
Weight, kg	89.97 ± 0.54	78.56 ± 0.70	88.64 ± 0.72	93.19 ± 0.87	101.85 ± 0.98	< 0.0001
Total testosterone, ng/dl	420.22 ± 4.29	508.01 ± 7.59	414.83 ± 6.51	401.91 ± 7.91	340.68 ± 6.51	< 0.0001
Age group, %						< 0.0001
20-40y	37.61(0.02)	68.60(2.19)	37.27(1.93)	24.72(1.63)	14.60(2.05)	
40-60y	37.30(0.02)	26.19(2.02)	45.64(2.22)	44.80(1.96)	32.41(1.83)	
> 60y	25.09(0.02)	5.21(0.98)	17.09(1.87)	30.47(1.87)	52.99(1.90)	
Race, %						< 0.0001
Mexican American	9.12(0.01)	5.49(0.98)	10.03(1.64)	11.10(1.54)	10.23(2.23)	
Non-Hispanic White	67.80(0.05)	63.25(3.07)	68.05(2.51)	65.97(2.98)	74.99(2.73)	
Non-Hispanic Black	9.53(0.01)	16.86(2.03)	7.71(1.09)	7.94(1.06)	4.61(0.77)	
Other Race	13.55(0.01)	14.40(1.52)	14.21(1.43)	14.99(1.80)	10.18(1.17)	
Education, %						< 0.001
Less than high school	14.16(0.01)	10.67(1.15)	12.03(1.34)	16.44(1.83)	18.47(2.05)	
High school	22.82(0.02)	20.55(1.72)	23.08(1.94)	23.96(1.70)	23.98(2.18)	
More than high school	63.03(0.04)	68.77(2.17)	64.89(2.56)	59.60(2.26)	57.55(2.74)	
BMI, %						< 0.0001
Normal (< 25 kg/m^2^)	25.22(0.01)	56.31(2.05)	20.73(1.38)	12.80(1.46)	6.63(0.98)	
Overweight (25–30 kg/m^2^)	38.13(0.02)	34.97(2.29)	48.50(2.54)	39.65(2.10)	27.70(1.55)	
Obese (> 30 kg/m^2^)	36.65(0.02)	8.72(1.20)	30.77(2.29)	47.54(2.42)	65.67(1.60)	
PIR, %						0.0343
<1	12.92(0.01)	14.79(1.72)	10.43(0.99)	12.02(1.20)	14.66(2.13)	
≥1	87.08(0.05)	85.21(1.72)	89.57(0.99)	87.98(1.20)	85.34(2.13)	
Marital status, %						< 0.0001
Solitude	32.11(0.01)	42.85(2.42)	26.60(1.56)	27.10(1.74)	31.30(1.30)	
Cohabitation	67.89(0.04)	57.15(2.42)	73.40(1.56)	72.90(1.74)	68.70(1.30)	
Smoke, %						< 0.0001
Never	50.18(0.03)	59.91(2.17)	53.77(2.08)	45.83(1.97)	38.80(2.86)	
Former	29.46(0.02)	16.40(1.80)	26.64(1.78)	34.02(2.14)	43.71(2.38)	
Current	20.36(0.01)	23.69(1.69)	19.59(1.59)	20.16(1.63)	17.49(1.42)	
Alcohol, %						0.5641
No	8.12(0.01)	9.20(1.96)	8.49(1.68)	7.37(1.28)	7.17(0.94)	
Yes	91.88(0.04)	90.80(1.96)	91.51(1.68)	92.63(1.28)	92.83(0.94)	
Hypertension, %						< 0.0001
No	61.12(0.03)	81.67(1.75)	64.80(1.99)	53.85(1.97)	39.73(2.34)	
Yes	38.88(0.02)	18.33(1.75)	35.20(1.99)	46.15(1.97)	60.27(2.34)	
Diabetes, %						< 0.0001
No	74.60(0.04)	92.47(1.01)	81.03(1.79)	67.33(2.02)	53.09(2.20)	
Prediabetes	9.66(0.01)	4.61(0.80)	9.18(1.28)	12.48(1.61)	13.26(1.28)	
Yes	15.75(0.01)	2.91(0.63)	9.79(1.28)	20.19(1.86)	33.66(2.12)	
Testosterone deficiency, %						< 0.0001
No	74.46(0.03)	88.33(1.39)	78.22(1.59)	71.20(1.76)	56.69(2.85)	
Yes	25.54(0.02)	11.67(1.39)	21.78(1.59)	28.80(1.76)	43.31(2.85)	

**Abbreviations**: WWI: weight-adjusted waist index; BMI, body mass index; WC, waist circumference; PIR, ratio of family income to poverty

Weighted mean ± standard error for continuous variables: comparisons were performed by the weighted linear regression model

Weighted percentage for categorical variable: comparisons were performed by the weighted chi-square test

### Associations of WWI with testosterone level and testosterone deficiency

To better understand the relationships between the WWI, BMI, WC, and weight with total testosterone levels and the risk of TD, we conducted detailed multivariable regression analyses. The results are presented in Table S1. Firstly, the fully adjusted linear regression analysis results showed that each unit increase in BMI, WC, and weight corresponded to decreases in total testosterone levels of -7.79 (-10.49, -5.09), -3.74 (-4.53, -2.95), and − 1.82 (-2.40, -1.24), respectively. Similarly, when these variables were converted into quartiles, participants in Q4 compared to Q1 showed total testosterone level decreases of -106.87 (-168.44, -71.30), -106.12 (-164.29, -102.94), and − 74.19 (-104.28, -44.10), respectively. Secondly, the fully adjusted logistic regression analysis indicated that each unit increase in BMI, WC, and weight corresponded to increased risks of TD of 1.12 (1.10, 1.15), 1.02 (1.01, 1.06), and 1.01 (1.00, 1.03), respectively. When these variables were converted into quartiles, participants in Q4 compared to Q1 showed risks of TD as 2.47 (1.32, 6.87), < 0.0001; 2.31 (2.11, 4.62), < 0.0001; and 2.61 (1.61, 4.22), 0.001, respectively. Moreover, in both unadjusted and minimally adjusted models, these results remained statistically significant. Additionally, the trend tests for the quartile variables showed values less than 0.05, indicating significant trends. These associations with total testosterone levels and the risk of TD are all weaker compared to those with WWI. Table 2 displays the associations of WWI with total testosterone level and risk of TD. Weighted linear regression analyses demonstrated consistent negative associations between WWI and total testosterone level in all models (non-adjusted model: β=-80.94, 95%CI: -87.90, -73.99, *P* < 0.0001; Model 1: β=-96.67, 95%CI: -105.99, -87.36, *P* < 0.0001; Model 2: β=-61.41, 95%CI: -72.53, -50.29, *P* < 0.0001). This negative association remained statistically significant across all models when we grouped the continuous WWI into quartiles (Q4, Q3, and Q2 compared to Q1; all *P* < 0.0001).

**Table 2:** The association between WWI and testosterone level from NHANES 2013–2016, weighted

WWI	Non-adjusted model	Model 1	Model 2
**Total testosterone (ng/dl)-β (95%CI) p-value**			
Continuous	-80.94 (-87.90, -73.99), < 0.0001	-96.67 (-105.99, -87.36), < 0.0001	-61.41( -72.53, -50.29), < 0.0001
Quartile 1	Reference	Reference	Reference
Quartile 2	-93.18 (-113.23, -73.12), < 0.0001	-101.43 (-122.50, -80.35), < 0.0001	-57.21 ( -78.23, -36.19), < 0.0001
Quartile 3	-106.1 (-126.99, -85.20), < 0.0001	-121.59 (-142.03, -101.16), < 0.0001	-62.23 ( -82.74, -41.72), < 0.0001
Quartile 4	-167.33 (-185.03, -149.64), < 0.0001	-189.64 (-212.11, -167.16), < 0.0001	-115.4 (-142.34, -88.45), < 0.0001
**P for trend**	< 0.0001	< 0.0001	< 0.0001
**Testosterone deficiency-OR (95% CI) p-value**			
Continuous	2.33(1.97,2.76), < 0.0001	2.67(2.21,3.23), < 0.0001	1.88 (1.47,2.39), < 0.0001
Quartile 1	Reference	Reference	Reference
Quartile 2	2.11 (1.59, 2.79), < 0.0001	2.31 (1.73, 3.08), < 0.0001	1.53 (1.14, 2.06), 0.01
Quartile 3	3.06 (2.33, 4.02), < 0.0001	3.57 (2.70, 4.71), < 0.0001	2.00 (1.49, 2.67), < 0.001
Quartile 4	5.78 (3.93, 8.51), < 0.0001	7.20 (4.75, 10.92), < 0.0001	3.38 (2.10, 5.44), < 0.001
**P for trend**	< 0.0001	< 0.0001	< 0.001

**Abbreviations**: WWI: weight-adjusted waist index; BMI, body mass index; WC, waist circumference; PIR, ratio of family income to poverty; OR, odds ratio; CI, confidence interval; β: effect size for linear regression

**Notes**: Non-adjusted model with no covariates adjusted; Model 1 was adjusted for age, race, education, and PIR; Model 2 was furtherly adjusted for BMI, marital status, smoking, alcohol consumption, hypertension, and diabetes based on Model 1

**Fig. 2:**
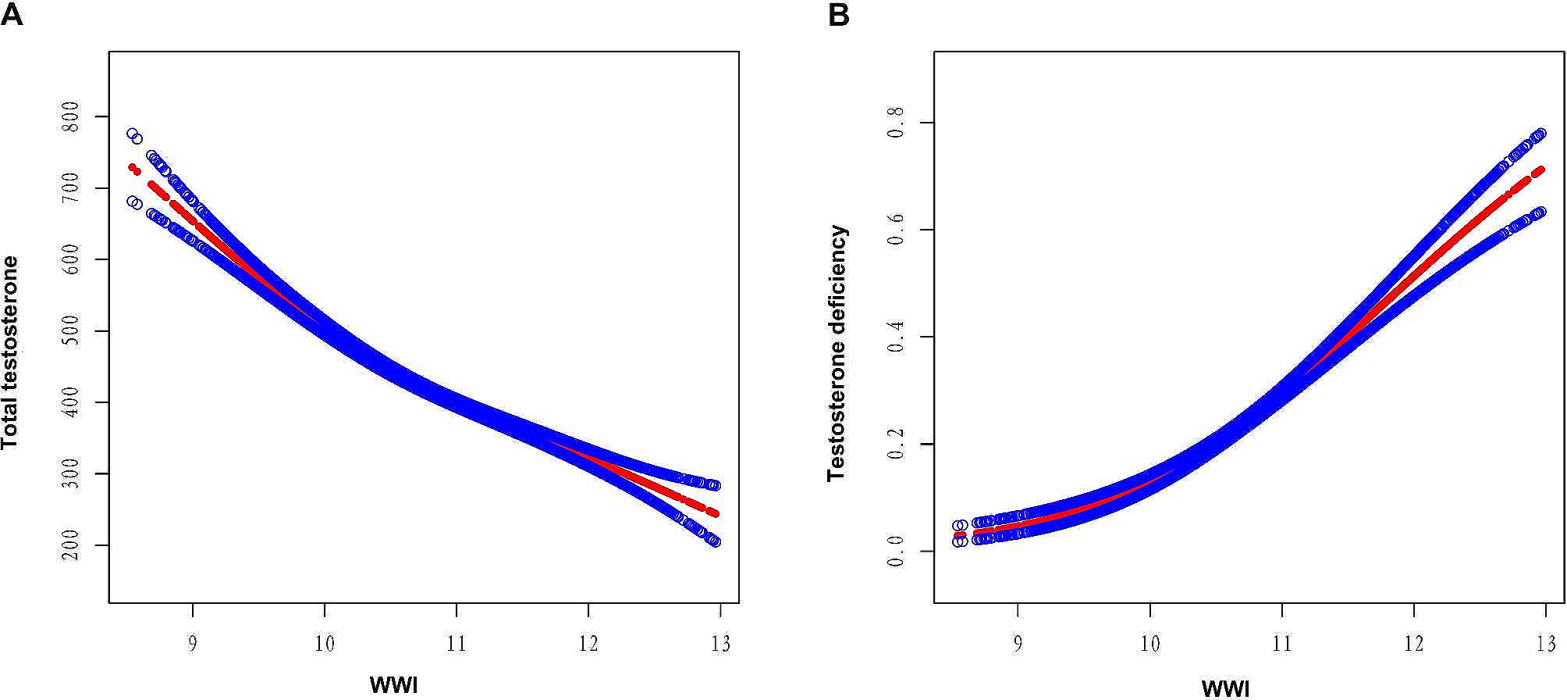
Graphics of smooth curve fittings between WWI and total testosterone level and testosterone deficiency. Blue bands represent the 95% CI from the fit. The solid rad line represents the smooth curve fit between variables

Subsequently, weighted logistic regression analyses were performed to investigate the association between WWI and occurrence of TD. The results indicated a positive association between WWI and risk of TD in all models (non-adjusted model: OR = 2.33, 95%CI: 1.97, 2.76, *P* < 0.0001; Model 1: OR = 2.67, 95%CI: 2.21, 3.23, *P* < 0.0001; Model 2: OR = 1.88, 95%CI: 1.47, 2.39, *P* < 0.0001). Participants in WWI quartile 4 had a significantly 338% higher risk of TD than those in WWI quartile 1, with all potential covariates adjusted (Model 2, OR = 3.38, 95% CI: 2.10, 5.44, *P* < 0.001, P for trend < 0.001). Both linear and logistic regression analyses indicated that the strength of the association between WWI and total testosterone levels and the risk of TD is significantly higher than that of traditional obesity assessment indicators such as BMI, WC, and weight. Figure 2 displays smooth curve fittings investigating the nonlinear associations of the WWI with total testosterone level and risk of TD. These associations were generally stable and linear. Specifically, there was a negative linear correlation between WWI and total testosterone level (Fig. 2A), while the risk of testosterone deficiency exhibited a positive linear correlation with WWI (Fig. 2B).

### Subgroup analysis

Table 3 lists the subgroup analyses stratified by age, BMI, diabetes, and hypertension, considering WWI as a continuous variable. The negative association between WWI and total testosterone level remained significant among all subgroups without interactions (all p for interaction > 0.05). Similarly, the positive association between WWI and risk of TD also remained significant among all subgroups without interactions (all p for interaction > 0.05). When the WWI was divided into quartiles, the results of subgroup analyses were listed in Table 4. The negative relationship between total testosterone levels and WWI quartiles remained consistent when comparing Q4 with Q1. The positive relationship between the risk of TD and WWI quartiles remained stable in most subgroups, except for the BMI subgroup. In BMI subgroup, the positive association was only significant among overweight and obese participants with a BMI ≥ 25 kg/m^2^ (Overweight: Q4 vs. Q1: OR = 3.66, 95%CI: 1.73, 7.75, *P* < 0.0001; Obese: Q4 vs. Q1: OR = 2.77, 95%CI: 1.07, 7.16, *P* = 0.039). All interaction terms indicated no dependence on theses subgroup for these associations (all p for interaction > 0.05).

**Table 3:** Subgroup analysis of the association between continuous WWI and testosterone level as well as testosterone deficiency, weighted

Subgroup	Cases/participants	Total testosterone (ng/dl)-β (95%CI) *p*-value		Testosterone deficiency-OR (95% CI) *p*-value	
		β (95%CI)	*P* for interaction	OR (95%CI)	*P* for interaction
Age group			0.118		0.173
20-40y	1435/4099	-72.84 ( -87.96, -57.73), < 0.0001		2.28 (1.61, 3.23), < 0.001	
40-60y	1354/4099	-55.64 ( -78.42, -32.86), < 0.001		1.72 (1.20, 2.47), 0.006	
>60y	1310/4099	-55.11 ( -84.69, -25.52), 0.01		1.74 (1.09, 2.76), 0.023	
BMI			0.139		0.915
Normal (< 25 kg/m^2^)	1115/4099	-78.63 (-111.14, -46.11), < 0.001		1.74 (1.05, 2.89), 0.003	
Overweight (25-<30 kg/m^2^)	1550/4099	-62.46 (-79.37, -45.55), < 0.0001		1.93 (1.24, 3.00), 0.006	
Obese (≥ 30 kg/m^2^)	1434/4099	-51.54 (-77.35, -25.73), < 0.001		1.95 (1.40, 2.70), < 0.001	
Smoke			0.517		0.069
Never	1946/4099	-59.83(-73.11, -46.55), < 0.0001		2.24(1.76,2.85), < 0.001	
Former	1222/4099	-67.50(-88.35, -46.65), < 0.0001		1.98(1.33,2.95), 0.002	
Current	931/4099	-60.37(-83.36, -37.37), < 0.0001		1.29(0.80,2.08), 0.28	
DM			0.086		0.899
No	2686/4099	-62.90 ( -75.82, -49.98), < 0.0001		1.89 (1.47, 2.43), < 0.001	
Borderline	406/4099	-41.09 ( -78.68, -3.52), 0.034		2.36 (1.12, 5.00), 0.027	
Yes	825/4099	-67.34 (-101.60, -33.08), < 0.001		1.86 (1.10, 3.15), 0.025	
Hypertension			0.184		0.420
No	2368/4099	-63.82 ( -78.42, -49.23), < 0.0001		1.98 (1.50, 2.61), < 0.001	
Yes	1731/4099	-57.45 ( -81.21, -33.70), < 0.001		1.79 (1.24, 2.60), 0.005	

**Abbreviations**: WWI: weight-adjusted waist index; BMI, body mass index; WC, waist circumference; PIR, ratio of family income to poverty; DM: diabetes mellitus; OR, odds ratio; CI, confidence interval; β: effect size for linear regression

**Notes**: All subgroup analyses were performed in Model 2 with age, race, education, PIR, BMI, marital status, smoking, alcohol consumption, hypertension, and diabetes adjusted

**Table 4:** Subgroup analysis of the association between WWI quartiles and testosterone level as well as testosterone deficiency, weighted

Subgroup	Quartile 1	Quartile 2	Quartile 3	Quartile 4	*P* for trend	*P* for interaction
**Total testosterone (ng/dl)-β (95%CI)** ***p*** **-value**						
Age group						0.140
20-40y	Ref	-50.70( -79.45, -21.94), 0.002	-97.69(-124.70, -70.68), < 0.001	-124.27(-153.78, -94.75), < 0.001	< 0.0001	
40-60y	Ref	-56.06( -94.11, -18.01), 0.007	-39.71( -83.89, 4.47), 0.074	-105.64(-150.10, -61.17), < 0.001	< 0.001	
>60y	Ref	-60.49(-124.00, 3.01), 0.060	-68.89(-131.79, -6.00), 0.034	-128.66(-200.76, -56.57), 0.002	< 0.001	
BMI						0.450
Normal (< 25 kg/m^2^)	Ref	-65.48(-116.22, − 14.73), 0.015	-54.14(-115.91, 7.62), 0.081	-135.71(-203.04, -68.38), < 0.001	0.003	
Overweight (25-<30 kg/m^2^)	Ref	-62.03( -94.60, -29.45), 0.001	-63.08( -96.07, -30.09), 0.001	-112.29(-151.22, -73.36), < 0.001	< 0.0001	
Obese (≥ 30 kg/m^2^)	Ref	-32.54( -71.64, 6.56), 0.095	-45.96( -82.05, -9.87), 0.016	-104.22(-155.21, -53.23), < 0.001	< 0.0001	
Smoke						0.258
Never	Ref	-49.09( -77.06, -21.12), 0.002	-71.49( -98.37, -44.60), < 0.0001	-106.7(-137.70, -75.71), < 0.0001	< 0.0001	
Former	Ref	-80.84(-137.90, -23.77), 0.009	-68.99(-116.88, -21.10), 0.009	-151.81(-206.32, -97.31), < 0.001	< 0.0001	
Current	Ref	-51.4( -90.88, -11.92), 0.015	-43.98( -78.28, -9.68), 0.016	-93.52(-143.85, -43.20), 0.002	0.002	
DM						0.095
No	Ref	-57.93( -81.20, -34.67), < 0.001	-70.35( -95.73, -44.97), < 0.001	-114.11(-143.22, -85.01), < 0.001	< 0.0001	
Borderline	Ref	-2.96( -62.61, 56.68), 0.916	21.88( -52.56, 96.31), 0.536	-68.22(-134.46, -1.98), 0.044	0.043	
Yes	Ref	-61.38(-114.88, -7.89), 0.028	-60.96(-101.53, -20.38), 0.006	-126.37(-185.73, -67.01), < 0.001	0.001	
Hypertension						0.141
No	Ref	-59.87( -90.00, -29.73), < 0.001	-72.87( -97.46, -48.28), < 0.001	-112.50(-145.94, -79.06), < 0.001	< 0.0001	
Yes	Ref	-38.16( -76.51, 0.18), 0.052	-35.20( -69.89, -0.52), 0.047	-106.73(-153.98, -59.48), < 0.001	< 0.001	
**Testosterone deficiency-OR (95% CI) p-value**						
Age group						0.127
20-40y	Ref	1.05 (0.62, 1.80), 0.841	2.72 (1.50, 4.91), 0.003	4.28 (2.35, 7.78), < 0.001	< 0.0001	
40-60y	Ref	1.80 (0.95, 3.41), 0.068	1.49 (0.77, 2.87), 0.213	3.18 (1.43, 7.08), 0.008	0.018	
>60y	Ref	2.62 (0.51, 13.50), 0.225	3.09 (0.68, 14.04), 0.131	4.86 (0.90, 26.23), 0.063	0.028	
BMI						0.114
Normal (< 25 kg/m^2^)	Ref	1.76 (0.69, 4.47), 0.214	1.06 (0.45, 2.56), 0.874	2.21 (0.88, 5.54), 0.085	0.228	
Overweight (25–30 kg/m^2^)	Ref	1.70 (0.99, 2.92), 0.052	1.97 (0.97, 4.01), 0.059	2.77 (1.07, 7.16), 0.038	0.039	
Obese (> 30 kg/m^2^)	Ref	1.14 (0.60, 2.15), 0.671	1.98 (0.99, 3.94), 0.052	3.66 (1.73, 7.75), 0.002	< 0.0001	
Smoke						0.069
Never	Ref	1.68(1.11,2.52), 0.018	2.94(2.07,4.18), < 0.0001	4.55(2.91,7.12), < 0.0001	< 0.0001	
Former	Ref	1.94(0.92, 4.09), 0.078	1.79(0.90, 3.58), 0.092	4.32(1.81,10.33), 0.003	0.002	
Current	Ref	0.75(0.31, 1.81), 0.492	0.84(0.42, 1.69), 0.602	1.11(0.41, 3.00), 0.827	0.703	
DM						0.616
No	Ref	1.41 (1.00, 1.98), 0.051	2.00 (1.44, 2.78), < 0.001	3.28 (1.86, 5.81), < 0.001	< 0.001	
Borderline	Ref	2.56 (0.50, 13.08), 0.235	3.27 (0.70, 15.42), 0.122	6.70 (1.35, 33.24), < 0.024	0.015	
Yes	Ref	5.50 (1.27, 23.86), 0.026	4.79 (1.09, 21.06), 0.040	9.13 (2.06, 40.50), 0.007	0.008	
Hypertension						0.731
No	Ref	1.57 (1.06, 2.32), 0.027	2.12 (1.40, 3.20), 0.002	3.95 (2.47, 6.31), < 0.0001	< 0.0001	
Yes	Ref	1.33 (0.68, 2.59), 0.372	1.66 (0.90, 3.05), 0.097	2.72 (1.30, 5.68), 0.012	0.008	

**Abbreviations**: WWI: weight-adjusted waist index; BMI, body mass index; WC, waist circumference; PIR, ratio of family income to poverty; DM: diabetes mellitus; OR, odds ratio; CI, confidence interval; β: effect size for linear regression

**Notes**: The continuous WWI was categorized into quartiles (Quartile 1, Quartile 2, Quartile 3, and Quartile 4). All subgroup analyses were performed in Model 2 with age, race, education, PIR, BMI, marital status, smoking, alcohol consumption, hypertension, and diabetes adjusted

## Discussion

The present study investigated the association between WWI and total testosterone level as well as risk of TD using NHANES, a representative national data set. Our results showed a strong and negative association between the WWI and total testosterone level. A positive association between WWI and risk of TD was also identified in all three models. Subgroup analysis and interaction test showed that these associations between WWI and total testosterone level as well as risk of TD were stable. To our knowledge, this is the first large-scale study to investigate the relationship between WWI and total testosterone level as well as risk of TD using nationally representative data.

There is a strong link between obesity and TD, and several studies have shown a significant negative correlation between the degree of obesity and total testosterone, free testosterone, and bioavailable testosterone (free and bound to albumin) [32–36], an association that is stable across age groups [37]. Several other clinical studies have also reported a negative correlation between obesity level and total testosterone [16, 38–41], which was not affected by metabolic syndrome [42]. Specifically, several studies have demonstrated a relationship between measures of obesity and testosterone, with plasma total testosterone, free testosterone, and SHBG levels negatively correlating with WC [35, 36], and BMI and WC associated with testosterone deficiency in males with diabetes [39, 40]. Common comorbidities of male obesity include hypogonadism (low testosterone levels and accompanying signs and symptoms) [43, 44]. In addition, a meta-analysis showed that testosterone levels in men can be significantly elevated after weight loss through exercise, diet, or bariatric surgery [45].

Currently, several explanations for the association between obesity and TD have been proposed. First, obesity can directly affect testosterone levels. Adipocytes express high levels of aromatase, which enzymatically cleaves testosterone to estrogen, reducing testosterone levels in the body. At the same time, estrogen can negatively feedback on the hypothalamic-pituitary (HP) axis, inhibiting gonadotropin-releasing hormone (GnRH) and subsequently luteinizing hormone (LH), which leads to a decrease in testosterone levels, and this creates a negative obesity-hypogonadism cycle [46]. Additionally, adipocytes may produce pro-inflammatory factors to regulate testosterone production. Elevated adiposity is strongly associated with increased plasma pro-inflammatory cytokines [47, 48], and related studies have found that pro-inflammatory cytokines are significantly higher and anti-inflammatory cytokines are lower in overweight and obese individuals than in healthy lean individuals [49]. Obesity particularly increases the pro-inflammatory factors interleukin-1 (IL-1), IL-6, and tumor necrosis factor-alpha (TNFα), which in turn stimulate the liver to produce other cytokines [50]. Inflammatory factors such as IL-6 and TNFα also have a role in inhibiting hypothalamic adrenocorticotropic hormone secretion, which reduces testosterone levels [14]. The alternative explanation is that a fat-derived hormone, leptin, plays a role between obesity and testosterone. Increased body fat in obese individuals dramatically raises leptin levels, which suppresses central kisspeptin levels, leading to decreased hypothalamic GnRH levels, reduced pituitary LH/FSH levels, and ultimately, decreased testosterone secretion in the testes [14, 51, 52]. In addition, elevated leptin levels can directly inhibit testosterone production in testicular cells, thus further reducing testosterone levels [51].

A number of metrics have been used as measures of obesity to predict correlations with associated health risks, such as body weight, BMI, WC, and waist-to-hip ratio, but these parameters do not comprehensively characterize obesity, and there are variations in the strength of prediction in different diseases [53–55]. In fact, it is not obesity that is associated with testosterone levels in men, but rather the amount of fat, especially where it is distributed. Studies have reported a negative correlation between testosterone levels and body fat, with hypogonadal men having an increase in fat mass, abdominal or central obesity, despite a decrease in lean body mass [56–59]. Traditional indicators of obesity, such as BMI, are unable to distinguish between lean and fat body weights, and their accuracy has been consistently questioned in recent years [60–62]. Therefore, studies investigating relevant metrics to measure aspects of body composition may more accurately reveal the relationship between obesity and testosterone. In recent years, visceral fat has been proposed to more accurately reflect metabolic dysfunction, and it is often associated with abdominal obesity [19]. WWI is an indicator based on WC and weight calculation, mainly reflecting the actual situation of central obesity. Central obesity is mainly caused by the accumulation of visceral fat, which is closely associated with endocrine and metabolic diseases [63, 64]. Moreover, researches also indicated strong relationship between TD and visceral adiposity [14]. Thus, compared to other obesity measures, the WWI primarily reflects the accumulation of visceral fat [28]. Given the relationship between adipose tissue and testosterone levels, WWI may be a more detailed reflection of the association between obesity and TD, and our study demonstrated a close association between WWI and TD. This study provides new ideas for future studies exploring the relationship between obesity and total testosterone level as well as TD. Further studies could extend the research population to children to investigate the impact of WWI on testosterone levels in children. Additionally, future research could evaluate the effectiveness of obesity interventions in improving TD.

Several results from the subgroup analyses merit further discussion, including those from different BMI subgroups, smoking status, and history of hypertension and diabetes. We found that among obese participants (BMI ≥ 30 kg/m²), the decrease in total testosterone levels associated with each unit increase in WWI was less pronounced compared to other groups, and the risk of TD was also lower. This may be because participants with higher BMI are more likely to engage in physical exercise and dietary control to reduce their weight, which can increase testosterone levels, thereby weakening the observed association. For the smoking status subgroup, our results were consistent with previous related studies [65]. Among smokers, the decrease in total testosterone levels per unit increase in WWI was less pronounced, and the risk of testosterone deficiency did not reach statistical significance. The possible reasons are as follows: exposure to nicotine and other toxins in smokers can directly impair testicular function, leading to decreased testosterone levels [66]. Additionally, smoking competes with eating for rewards, potentially reducing food intake and consequently obesity among smokers [67]. This might explain the weakened relationship between WWI and testosterone levels and the risk of testosterone deficiency in the smoking group. As for participants with diabetes, they are at a higher risk of TD compared to non-diabetics. This is due to the fact that, in addition to obesity, diabetic patients often have insulin resistance, hyperuricemia, and hyperlipidemia, all of which are significant risk factors for TD. Therefore, these factors further increase the risk of TD in diabetic patients [68]. Contrary to the above, we found that hypertensive participants are at a lower risk of TD compared to non-hypertensives. There is currently no evidence to suggest that hypertension causes TD, but TD is a significant risk factor for hypertension [69]. This implies that hypertensive patients might supplement with testosterone to improve the effectiveness of hypertension treatment, thereby mitigating the association between WWI and risk of TD in these participants. However, we must remember that these subgroup explanations need further research for validation.

There are some limitations in our study. First, since this study is cross-sectional, causality cannot be explored. Second, despite adjusting for some relevant confounders, we were unable to completely exclude fluctuations in other confounding variables, and therefore our results should be treated with caution. Third, the wide CI observed in the subgroup analyses may be due to the small sample size within each stratum, suggesting that the apparent interaction with age might be influenced by the insufficient number of participants in each age group. Fourth, the study lacks data on free testosterone and bioavailable testosterone, limiting the comprehensiveness of our findings. Additionally, due to the limitations of the NHANES database, our diagnosis of TD relies solely on a total testosterone level below 300 ng/dL without considering the symptoms and/or signs associated with TD. It is important to recognize that TD is not merely a biochemical diagnosis but also involves a spectrum of symptoms such as depression and decreased energy. Finally, the NHANES database represents only populations from the US. Whether the relationship between WWI and testosterone exists in other national or regional populations needs to be verified by more studies. However, this study also has several strengths worth highlighting. Firstly, this is the largest study to date and the first to investigate the relationship between WWI and both testosterone levels and the risk of TD. The large sample size ensured that we could perform analyses across different subgroups, thereby increasing the robustness of our results. Secondly, the high-quality NHANES data allowed us to include potential covariates that might influence the relationship between WWI and testosterone levels. Additionally, we compared the strength of the associations between WWI, BMI, WC, and weight with total testosterone levels and the risk of TD, thereby enriching the data and expanding its clinical applicability. Lastly, we carefully considered weighting in our data analysis, enhancing the representativeness of our sample and the reliability of our findings.

## Conclusion

Analyzing a nationally representative sample, our study revealed a negative association between WWI and total testosterone level, coupled with a positive association between WWI and risk of TD. Importantly, these associations remained stable across participants with different age, BMI, hypertension, and diabetes statuses. These findings suggest that WWI could be a valuable tool in public health and clinical practice for early identification and intervention in high-risk populations. Incorporating WWI measurements into routine clinical evaluations could help healthcare providers better assess central obesity and its impact on hormonal health, improving patient outcomes. However, further prospective studies are imperative in the future to validate our findings and delve into the underlying mechanisms of these associations.

### Electronic Supplementary Material

Below is the link to the electronic supplementary material.

Supplementary Material 1

## Data Availability

The survey data are publicly available on the internet for data users and researchers throughout the world (www.cdc.gov/nchs/nhanes/). Further R code and related materials could be requested from the corresponding author upon reasonable inquiry.

## Abbreviations

BMI: Body Mass Index
CI: Confidence Interval
CT: Computed Tomography
DXA: Dual-Energy X-ray Absorptiometry
GnRH: Gonadotropin-Releasing Hormone
HP: Hypothalamic-Pituitary
HPGA: Hypothalamic-Pituitary-Gonadal Axis
ID-LC–MS/MS: Isotope Dilution Liquid Chromatography Tandem Mass Spectrometry
IL-1: Interleukin-1
IL-6: Interleukin-6
LH: Luteinizing Hormone
MRI: Magnetic Resonance Imaging
NCHS: National Centre for Health Statistics
NIST: National Institute for Standards and Technology
NHANES: National Health and Nutrition Examination Survey
OGTT: Oral Glucose Tolerance Test
OR: Odds Ratio
PIR: Poverty Income Ratio
SE: Standard Error
TD: Testosterone Deficiency
TNFα: Tumor Necrosis Factor-Alpha
WC: Waist Circumference
WWI: Weight-Adjusted-Waist Index

## References

[1] Halpern JA, Brannigan RE (2019). Testosterone Deficiency. JAMA.

[2] Muller MN (2017). Testosterone and reproductive effort in male primates. Horm Behav.

[3] Iliescu R, Reckelhoff JF (2006). Testosterone and vascular reactivity. Clin Sci (Lond).

[4] Elagizi A, Köhler TS, Lavie CJ (2018). Testosterone and Cardiovascular Health. Mayo Clin Proc.

[5] Dimopoulou C, Goulis DG, Corona G, Maggi M (2018). The complex association between metabolic syndrome and male hypogonadism. Metabolism.

[6] Cherrier MM (2005). Androgens and cognitive function. J Endocrinol Invest.

[7] Traish AM, Miner MM, Morgentaler A, Zitzmann M (2011). Testosterone deficiency. Am J Med.

[8] Aversa A, Morgentaler A (2015). The practical management of testosterone deficiency in men. Nat Rev Urol.

[9] Wu S, Wu Y, Fang L, Zhao J, Cai Y, Xia W (2023). A negative association between triglyceride glucose-body mass index and testosterone in adult males: a cross-sectional study. Front Endocrinol (Lausanne).

[10] Trends in adult (2016). Body-mass index in 200 countries from 1975 to 2014: a pooled analysis of 1698 population-based measurement studies with 19·2 million participants. Lancet.

[11] Ward ZJ, Bleich SN, Cradock AL, Barrett JL, Giles CM, Flax C, Long MW, Gortmaker SL (2019). Projected U.S. State-Level prevalence of adult obesity and severe obesity. N Engl J Med.

[12] Gurayah AA, Mason MM, Masterson JM, Kargi AY, Ramasamy R (2023). U-shaped association between prevalence of secondary hypogonadism and body mass index: a retrospective analysis of men with testosterone deficiency. Int J Impot Res.

[13] Ren Y, Wang B, Liu X, Li Z, Yuan W, Sun Y, Miao M (2014). Association between body fat distribution and androgen deficiency in middle-aged and elderly men in China. Int J Impot Res.

[14] Kelly DM, Jones TH (2015). Testosterone and obesity. Obes Rev.

[15] Grossmann M (2018). Hypogonadism and male obesity: focus on unresolved questions. Clin Endocrinol (Oxf).

[16] Glass AR, Swerdloff RS, Bray GA, Dahms WT, Atkinson RL (1977). Low serum testosterone and sex-hormone-binding-globulin in massively obese men. J Clin Endocrinol Metab.

[17] Deng C, Zhang Z, Li H, Bai P, Cao X, Dobs AS (2019). Analysis of cardiovascular risk factors associated with serum testosterone levels according to the US 2011–2012 National Health and Nutrition Examination Survey. Aging Male.

[18] Oliveros E, Somers VK, Sochor O, Goel K, Lopez-Jimenez F (2014). The concept of normal weight obesity. Prog Cardiovasc Dis.

[19] Thomas EL, Frost G, Taylor-Robinson SD, Bell JD (2012). Excess body fat in obese and normal-weight subjects. Nutr Res Rev.

[20] Wang ZY, Li BC, Xing JJ, Liu SY, Zhang TT, Xu AM, Wang ZJ (2023). Associations of waist circumference with sex steroid hormones among 4031 USA children and adolescents. Asian J Androl.

[21] Mahmoud I, Al-Wandi AS, Gharaibeh SS, Mohamed SA (2021). Concordances and correlations between anthropometric indices of obesity: a systematic review. Public Health.

[22] Yu L, Chen Y, Xu M, Li R, Zhang J, Zhu S, He Z, Chen M, Wang G (2023). Association of weight-adjusted-waist index with asthma prevalence and the age of first asthma onset in United States adults. Front Endocrinol (Lausanne).

[23] Park Y, Kim NH, Kwon TY, Kim SG (2018). A novel adiposity index as an integrated predictor of cardiometabolic disease morbidity and mortality. Sci Rep.

[24] Qin Z, Chang K, Yang Q, Yu Q, Liao R, Su B (2022). The association between weight-adjusted-waist index and increased urinary albumin excretion in adults: a population-based study. Front Nutr.

[25] Hu Q, Han K, Shen J, Sun W, Gao L, Gao Y (2023). Association of weight-adjusted-waist index with non-alcoholic fatty liver disease and liver fibrosis: a cross-sectional study based on NHANES. Eur J Med Res.

[26] Ke B, Sun Y, Dai X, Gui Y, Chen S (2023). Relationship between weight-adjusted waist circumference index and prevalence of gallstones in U.S. adults: a study based on the NHANES 2017–2020. Front Endocrinol (Lausanne).

[27] Han Y, Shi J, Gao P, Zhang L, Niu X, Fu N (2023). The weight-adjusted-waist index predicts all-cause and cardiovascular mortality in general US adults. Clin (Sao Paulo).

[28] Kim NH, Park Y, Kim NH, Kim SG (2021). Weight-adjusted waist index reflects fat and muscle mass in the opposite direction in older adults. Age Ageing.

[29] Kim JE, Choi J, Kim M, Won CW (2023). Assessment of existing anthropometric indices for screening sarcopenic obesity in older adults. Br J Nutr.

[30] Kim KJ, Son S, Kim KJ, Kim SG, Kim NH (2023). Weight-adjusted waist as an integrated index for fat, muscle and bone health in adults. J Cachexia Sarcopenia Muscle.

[31] Mulhall JP, Trost LW, Brannigan RE, Kurtz EG, Redmon JB, Chiles KA, Lightner DJ, Miner MM, Murad MH, Nelson CJ (2018). Evaluation and management of Testosterone Deficiency: AUA Guideline. J Urol.

[32] Allen NE, Appleby PN, Davey GK, Key TJ (2002). Lifestyle and nutritional determinants of bioavailable androgens and related hormones in British men. Cancer Causes Control.

[33] Gapstur SM, Gann PH, Kopp P, Colangelo L, Longcope C, Liu K (2002). Serum androgen concentrations in young men: a longitudinal analysis of associations with age, obesity, and race. The CARDIA male hormone study. Cancer Epidemiol Biomarkers Prev.

[34] Jensen TK, Andersson AM, Jørgensen N, Andersen AG, Carlsen E, Petersen JH, Skakkebaek NE (2004). Body mass index in relation to semen quality and reproductive hormones among 1,558 Danish men. Fertil Steril.

[35] Svartberg J, von Mühlen D, Schirmer H, Barrett-Connor E, Sundfjord J, Jorde R (2004). Association of endogenous testosterone with blood pressure and left ventricular mass in men. The Tromsø Study. Eur J Endocrinol.

[36] Svartberg J, von Mühlen D, Sundsfjord J, Jorde R (2004). Waist circumference and testosterone levels in community dwelling men. The Tromsø study. Eur J Epidemiol.

[37] Corona G, Mannucci E, Ricca V, Lotti F, Boddi V, Bandini E, Balercia G, Forti G, Maggi M (2009). The age-related decline of testosterone is associated with different specific symptoms and signs in patients with sexual dysfunction. Int J Androl.

[38] Laaksonen DE, Niskanen L, Punnonen K, Nyyssönen K, Tuomainen TP, Salonen R, Rauramaa R, Salonen JT (2003). Sex hormones, inflammation and the metabolic syndrome: a population-based study. Eur J Endocrinol.

[39] Kapoor D, Goodwin E, Channer KS, Jones TH (2006). Testosterone replacement therapy improves insulin resistance, glycaemic control, visceral adiposity and hypercholesterolaemia in hypogonadal men with type 2 diabetes. Eur J Endocrinol.

[40] Hackett G, Cole N, Bhartia M, Kennedy D, Raju J, Wilkinson P (2013). Testosterone replacement therapy with long-acting testosterone undecanoate improves sexual function and quality-of-life parameters vs. placebo in a population of men with type 2 diabetes. J Sex Med.

[41] Corona G, Rastrelli G, Maggi M (2013). Diagnosis and treatment of late-onset hypogonadism: systematic review and meta-analysis of TRT outcomes. Best Pract Res Clin Endocrinol Metab.

[42] Kaplan SA, Meehan AG, Shah A (2006). The age related decrease in testosterone is significantly exacerbated in obese men with the metabolic syndrome. What are the implications for the relatively high incidence of erectile dysfunction observed in these men?. J Urol.

[43] Rothdach AJ, Trenkwalder C, Haberstock J, Keil U, Berger K (2000). Prevalence and risk factors of RLS in an elderly population: the MEMO study. Memory and morbidity in Augsburg Elderly. Neurology.

[44] Dhindsa S, Miller MG, McWhirter CL, Mager DE, Ghanim H, Chaudhuri A, Dandona P (2010). Testosterone concentrations in diabetic and nondiabetic obese men. Diabetes Care.

[45] Corona G, Rastrelli G, Monami M, Saad F, Luconi M, Lucchese M, Facchiano E, Sforza A, Forti G, Mannucci E (2013). Body weight loss reverts obesity-associated hypogonadotropic hypogonadism: a systematic review and meta-analysis. Eur J Endocrinol.

[46] Cohen PG (1999). The hypogonadal-obesity cycle: role of aromatase in modulating the testosterone-estradiol shunt–a major factor in the genesis of morbid obesity. Med Hypotheses.

[47] Gelaye B, Revilla L, Lopez T, Suarez L, Sanchez SE, Hevner K, Fitzpatrick AL, Williams MA (2010). Association between insulin resistance and c-reactive protein among Peruvian adults. Diabetol Metab Syndr.

[48] Olson NC, Callas PW, Hanley AJ, Festa A, Haffner SM, Wagenknecht LE, Tracy RP (2012). Circulating levels of TNF-α are associated with impaired glucose tolerance, increased insulin resistance, and ethnicity: the insulin resistance atherosclerosis study. J Clin Endocrinol Metab.

[49] Patel C, Ghanim H, Ravishankar S, Sia CL, Viswanathan P, Mohanty P, Dandona P (2007). Prolonged reactive oxygen species generation and nuclear factor-kappab activation after a high-fat, high-carbohydrate meal in the obese. J Clin Endocrinol Metab.

[50] Schuster DP (2010). Obesity and the development of type 2 diabetes: the effects of fatty tissue inflammation. Diabetes Metab Syndr Obes.

[51] Isidori AM, Caprio M, Strollo F, Moretti C, Frajese G, Isidori A, Fabbri A (1999). Leptin and androgens in male obesity: evidence for leptin contribution to reduced androgen levels. J Clin Endocrinol Metab.

[52] Khodamoradi K, Khosravizadeh Z, Seetharam D, Mallepalli S, Farber N, Arora H (2022). The role of leptin and low testosterone in obesity. Int J Impot Res.

[53] Misra KB, Endemann SW, Ayer M (2006). Measures of obesity and metabolic syndrome in Indian americans in northern California. Ethn Dis.

[54] Eknoyan G (2008). Adolphe Quetelet (1796–1874)--the average man and indices of obesity. Nephrol Dial Transpl.

[55] Kawada T, Kuratomi Y, Kanai T, Suto S, Nishime A, Koizumi M, Nakano N (2009). Anthropometric obesity indices and metabolic syndrome in Japanese working men. Work.

[56] Phillips GB (1993). Relationship between serum sex hormones and the glucose-insulin-lipid defect in men with obesity. Metabolism.

[57] Couillard C, Gagnon J, Bergeron J, Leon AS, Rao DC, Skinner JS, Wilmore JH, Després JP, Bouchard C (2000). Contribution of body fatness and adipose tissue distribution to the age variation in plasma steroid hormone concentrations in men: the HERITAGE Family Study. J Clin Endocrinol Metab.

[58] Seidell JC, Björntorp P, Sjöström L, Kvist H, Sannerstedt R (1990). Visceral fat accumulation in men is positively associated with insulin, glucose, and C-peptide levels, but negatively with testosterone levels. Metabolism.

[59] Kapoor D, Malkin CJ, Channer KS, Jones TH (2005). Androgens, insulin resistance and vascular disease in men. Clin Endocrinol (Oxf).

[60] Javed A, Jumean M, Murad MH, Okorodudu D, Kumar S, Somers VK, Sochor O, Lopez-Jimenez F (2015). Diagnostic performance of body mass index to identify obesity as defined by body adiposity in children and adolescents: a systematic review and meta-analysis. Pediatr Obes.

[61] Johansen KL (2013). Obesity and body composition for transplant wait-list candidacy–challenging or maintaining the BMI limits?. J Ren Nutr.

[62] Dixon AE, Peters U (2018). The effect of obesity on lung function. Expert Rev Respir Med.

[63] Nomura K, Eto M, Kojima T, Ogawa S, Iijima K, Nakamura T, Araki A, Akishita M, Ouchi Y (2010). Visceral fat accumulation and metabolic risk factor clustering in older adults. J Am Geriatr Soc.

[64] Tatsumi Y, Nakao YM, Masuda I, Higashiyama A, Takegami M, Nishimura K, Watanabe M, Ohkubo T, Okamura T, Miyamoto Y (2017). Risk for metabolic diseases in normal weight individuals with visceral fat accumulation: a cross-sectional study in Japan. BMJ Open.

[65] Liu Q, Huang Y, Wang M, Jiang H, Zhang X. Association of lipid accumulation products with testosterone deficiency in adult American men: a cross-sectional study. Andrology 2022. 10.1111/andr.13355 36435978

[66] Linna MS, Ahotupa M, Irjala K, Pöllänen P, Huhtaniemi I, Mäkinen J, Perheentupa A, Vasankari TJ (2008). Smoking and low serum testosterone associates with high concentration of oxidized LDL. Ann Med.

[67] N’Goran AA, Studer J, Deline S, Henchoz Y, Baggio S, Mohler-Kuo M, Daeppen JB, Gmel G (2016). Bidirectional relationship between the body mass index and substance use in young men. Subst Abus.

[68] Andlib N, Sajad M, Kumar R, Thakur SC (2023). Abnormalities in sex hormones and sexual dysfunction in males with diabetes mellitus: a mechanistic insight. Acta Histochem.

[69] Wei D, Hou J, Liu X, Zhang L, Wang L, Liu P, Fan K, Zhang L, Nie L, Xu Q (2021). Interaction between testosterone and obesity on hypertension: a population-based cross-sectional study. Atherosclerosis.

