# Supplementary material for: Association between weight-adjusted waist index and testosterone deficiency in adult American men: findings from the national health and nutrition examination survey 2013–2016
Source: BMC Public Health. 2024 Jun 24;24:1683. doi: 10.1186/s12889-024-19202-5 (PMC11197353; doi:10.1186/s12889-024-19202-5)
Supplement: Supplementary file 1 — Supplementary Material 1 [file 12889_2024_19202_MOESM1_ESM.docx]

**Table S1.** The association between BMI, WC, as well as weight and testosterone level from NHANES 2013–2016, weighted.

| WWI | Non-adjusted model | Model 1 | Model 2 |
| --- | --- | --- | --- |
| **Total testosterone (ng/dl)-β (95%CI) p-value** | | | |
| BMI (continuous) | -11.14(-12.24, -10.05), < 0.0001 | -11.02(-12.14, -9.90), < 0.0001 | -7.79(-10.49, -5.09), < 0.0001 |
| BMI (categorized) |  |  |  |
| Quartile 1 | Reference | Reference | Reference |
| Quartile 2 | -86.77(-105.63, -67.90), < 0.0001 | -80.74( -99.61, -61.87), < 0.0001 | -31.8( -80.29, 16.69), 0.18 |
| Quartile 3 | -122.08(-140.93, -103.22), < 0.0001 | -117.09(-136.16, -98.02), < 0.0001 | -56.8(-105.67, -7.92), 0.03 |
| Quartile 4 | -189.89(-202.85, -176.93), < 0.0001 | -186.03(-199.21, -172.85), < 0.0001 | -106.87(-168.44, -71.30), < 0.001 |
| **P for trend** | < 0.0001 | < 0.0001 | < 0.0001 |
| WC (continuous) | -7.79(-10.49, -5.09), < 0.0001 | -4.56( -4.94, -4.19), < 0.0001 | -3.74( -4.53, -2.95), < 0.0001 |
| WC (categorized) |  |  |  |
| Quartile 1 | Reference | Reference | Reference |
| Quartile 2 | -88.37(-110.86, -65.87), < 0.0001 | -86.04(-108.74, -63.34), < 0.0001 | -57( -85.28, -28.71), < 0.001 |
| Quartile 3 | -136.93(-156.51, -117.36), < 0.0001 | -121.44(-156.79, -114.09), < 0.0001 | -58.66(-114.13, -53.19), < 0.0001 |
| Quartile 4 | -194.05(-209.69, -178.42), < 0.0001 | -163.83(-210.01, -157.65), < 0.0001 | -106.12(-164.29, -102.94), < 0.0001 |
| **P for trend** | < 0.0001 | < 0.0001 | < 0.0001 |
| Weight (continuous) | -3.07(-3.36, -2.77), < 0.0001 | -3.12( -3.41, -2.82), < 0.0001 | -1.82(-2.40, -1.24), < 0.0001 |
| Weight (categorized) |  |  |  |
| Quartile 1 | Reference | Reference | Reference |
| Quartile 2 | -59.55( -78.48, -40.62), < 0.0001 | -57.28( -76.50, -38.07), < 0.0001 | -15.85(-39.98, 8.29), 0.18 |
| Quartile 3 | -113.21(-130.94, -95.48), < 0.0001 | -111.45(-129.92, -92.97), < 0.0001 | -37.09(-61.98, -12.20), 0.01 |
| Quartile 4 | -172.27(-190.20, -154.35), < 0.0001 | -172.51(-190.44, -154.58), < 0.0001 | -74.19(-104.28, -44.10), < 0.001 |
| **P for trend** | < 0.0001 | < 0.0001 | < 0.0001 |
| **Testosterone deficiency-OR (95% CI) p-value** | | | |
| BMI (continuous) | 1.13(1.11,1.15), < 0.0001 | 1.13(1.11,1.15), < 0.0001 | 1.12(1.10,1.15), < 0.0001 |
| BMI (categorized) |  |  |  |
| Quartile 1 | Reference | Reference | Reference |
| Quartile 2 | 2.09(1.56,2.82), < 0.0001 | 2.00(1.49,2.68), < 0.0001 | 1.20(1.38,2.62), < 0.0001 |
| Quartile 3 | 2.74(2.11,3.56), < 0.0001 | 2.63(2.04,3.39), < 0.0001 | 1.95(1.79,3.19), < 0.0001 |
| Quartile 4 | 6.91(5.06,9.44), < 0.0001 | 5.79(4.99,9.24), < 0.0001 | 2.47(1.32,6.87), < 0.0001 |
| **P for trend** | < 0.0001 | < 0.0001 | < 0.001 |
| WC (continuous) | 1.05(1.04,1.06), < 0.0001 | 1.05(1.04,1.06), < 0.0001 | 1.02(1.01,1.06), < 0.0001 |
| WC (categorized) |  |  |  |
| Quartile 1 | Reference | Reference | Reference |
| Quartile 2 | 1.93(1.36, 2.73), < 0.001 | 1.87(1.32,2.64), 0.001 | 1.53(0.99,2.36), 0.05 |
| Quartile 3 | 3.31(2.35, 4.64), < 0.0001 | 3.23(2.27,4.57), < 0.0001 | 1.73(1.45,3.43), 0.002 |
| Quartile 4 | 7.30(5.30,10.03), < 0.0001 | 6.22(5.34,9.75), < 0.0001 | 2.31(2.11, 4.62), < 0.0001 |
| **P for trend** | < 0.0001 | < 0.0001 | < 0.0001 |
| Weight (continuous) | 1.03(1.03,1.04), < 0.0001 | 1.03(1.03,1.04), < 0.0001 | 1.01(1.00,1.03), < 0.0001 |
| Weight (categorized) |  |  |  |
| Quartile 1 | Reference | Reference | Reference |
| Quartile 2 | 1.69(1.22,2.34), 0.003 | 1.71(1.23,2.37), 0.003 | 1.27(0.77,2.11), 0.32 |
| Quartile 3 | 2.66(1.98,3.58), < 0.0001 | 2.78(2.06,3.73), < 0.0001 | 1.56(0.96,2.53), 0.07 |
| Quartile 4 | 5.70(4.30,7.56), < 0.0001 | 6.11(4.57,8.18), < 0.0001 | 2.61(1.61,4.22), 0.001 |
| **P for trend** | < 0.0001 | < 0.0001 | < 0.001 |

**Abbreviations:** WWI: weight-adjusted waist index; BMI, body mass index; WC, waist circumference; PIR, ratio of family income to poverty; OR, odds ratio; CI, confidence interval; β: effect size for linear regression.

**Notes:** Non-adjusted model with no covariates adjusted; Model 1 was adjusted for age, race, education, and PIR; Model 2 was furtherly adjusted for BMI, marital status, smoking, alcohol consumption, hypertension, and diabetes based on Model 1.
